# Best practices in bioinformatics training for life scientists

**DOI:** 10.1093/bib/bbt043

**Published:** 2013-06-25

**Authors:** Allegra Via, Thomas Blicher, Erik Bongcam-Rudloff, Michelle D. Brazas, Cath Brooksbank, Aidan Budd, Javier De Las Rivas, Jacqueline Dreyer, Pedro L. Fernandes, Celia van Gelder, Joachim Jacob, Rafael C. Jimenez, Jane Loveland, Federico Moran, Nicola Mulder, Tommi Nyrönen, Kristian Rother, Maria Victoria Schneider, Teresa K. Attwood

**Keywords:** bioinformatics, training, bioinformatics courses, training life scientists, train the trainers

## Abstract

The mountains of data thrusting from the new landscape of modern high-throughput biology are irrevocably changing biomedical research and creating a near-insatiable demand for training in data management and manipulation and data mining and analysis. Among life scientists, from clinicians to environmental researchers, a common theme is the need not just to use, and gain familiarity with, bioinformatics tools and resources but also to understand their underlying fundamental theoretical and practical concepts. Providing bioinformatics training to empower life scientists to handle and analyse their data efficiently, and progress their research, is a challenge across the globe. Delivering good training goes beyond traditional lectures and resource-centric demos, using interactivity, problem-solving exercises and cooperative learning to substantially enhance training quality and learning outcomes. In this context, this article discusses various pragmatic criteria for identifying training needs and learning objectives, for selecting suitable trainees and trainers, for developing and maintaining training skills and evaluating training quality. Adherence to these criteria may help not only to guide course organizers and trainers on the path towards bioinformatics training excellence but, importantly, also to improve the training experience for life scientists.

## INTRODUCTION

The hunger for bioinformatics training courses arose around the mid-‘80s, following the appearance of the first databases and software tools for analysing protein sequences and structures. Back then, there were no easy ways to disseminate such resources, and computer facilities in most life science laboratories were crude or non-existent. Novel data-distribution mechanisms had to be invented to ensure that the new resources were reaching their target audiences. The European Molecular Biology Network (EMBnet), for example, pioneered the distribution of the EMBL Data Library [1] from the EMBL in Heidelberg to national data centres holding government mandates to provide access to this and other bioinformatics databases and tools to their local communities [this model is now being adopted, on a much larger scale, by ELIXIR (http://www.elixir-europe.org/), a pan-European endeavour to provide a sustainable infrastructure for biological information, and which will generate even greater training needs] [2]. Such distribution networks solved many problems for data providers but demanded a certain level of end-user computational competence: first, to be able to login to a remote, centralized site; second, to be able to find and access the relevant databases or software tools on the remote system; and finally, to be able to export any results back to the local computer. The web did not exist, and most of these skills were the preserve of just a few self-taught “informaticians”, who were comfortable with arcane Internet communication protocols and search engines, such as Gopher [3], WAIS [4], Archie [5], HASSLE [6] and so on. Training courses became essential to allow life scientists to overcome the technical hurdles, their focus necessarily being on “how to access” bioinformatics tools and resources.

In the early ‘90s, the advent of intuitive graphical web browsers shifted the goal posts. For the first time, databases and software could be accessed instantaneously via customized web interfaces. These were designed to be as easy to use as possible, often “hiding” some of the more technical details and parameters behind “advanced” options that most users never dared to explore. However, as web technologies moved on, the database and software interfaces accreted greater degrees of functionality and, ironically, became harder and harder to use. Consequently, a new breed of training courses on “how to use” bioinformatics tools and resources was born.

The past decade has witnessed another shift: the industrialization of laboratory techniques has revolutionized the pace of data acquisition, computers are now standard laboratory equipment, and both the computational competence and computational requirements of life scientists have increased accordingly. The scale of data generation, today, is daunting—laboratory automation has made it possible to gather data first and to formulate hypotheses later. Indeed, such ‘data-driven science’ [3, 7] is now commonplace. Thus, more than ever before, researchers want to know, ‘How should I analyse my data?’, ‘How do I get the best out of this or that computational tool or resource?’, ‘What do my data mean?’ or even, ‘What is my hypothesis?’. Bioinformatics training courses are having to adapt to meet these new needs, but the pace of change has been swift, creating new challenges for course organizers and trainers, and ultimately also for trainees—how, for example, can they be certain of receiving the best, most excellent training?

## WHAT IS TRAINING EXCELLENCE?

The focus of training is the trainee. They ultimately judge training excellence not just in terms of how they perceive a particular training event, but also in terms of the impact this has on the development of their skills in the long-term. Excellence in training could be generally defined as the ability to deliver appropriate training in response to a particular demand, providing high-quality, up-to-date content and satisfying the expectations of trainees, of trainers and of the organization providing the training.

For several years, the Bioinformatics Training Network (BTN) [8] has provided a forum for bioinformatics trainers to share their experiences, to identify common challenges [9] and to agree on common working practices [10]. From these shared experiences and from round-table discussions on what can be understood by training excellence and how it might be achieved, four repeating themes have emerged that are generally applicable to the delivery of successful training for life scientists and beyond: (i) understanding the needs of trainees; (ii) ensuring that the training provided is suitable for a given audience; (iii) ensuring that a quality-assurance process is in place; and (iv) defining a sound organizational framework. These four aspects encompass many related facets; excelling in all is the key. From our collective perspectives and experiences, we prepared and made freely available, as a deliverable of the EU SLING project, an extensive document entitled, ‘*Bioinformatics training for life scientists: guidelines for best practice*’, based on what we believe ignites excellence in training: iterative performance of training events, assimilation of what did and did not work and feeding this information back in a dynamic feedback loop. Here, we present a summary of our discussions and invite all those in life science research and education to contribute to our on-going dialogue on how best to create a robust and sustainable foundation for bioinformatics learning, education and training.

### Identifying training needs

A training need arises when an individual is unable to perform a task adequately, or cannot perform it to a sufficiently high standard. Currently, significant training needs in the life sciences have arisen from the rapid advances in high-throughput data-production technologies, coupled with the volume and complexity of the data these are producing; the pace of change is so great that there is a growing lack of exposure to the tools and technologies for handling, retrieving, analysing and interpreting these data, and a dearth of understanding in how these might contribute to biological discovery. Courses addressing such needs are more likely to succeed if their target audiences are sufficiently specific to be able to narrow the focus to aspects that are relevant to the participants’ own research projects, to their level of background knowledge and to their technical experience with bioinformatics tools/resources. For example, from the technical standpoint, an important (but often over-looked) consideration is trainees’ familiarity with the Unix/Linux command line, R, etc—especially in courses that cover next-generation sequencing (NGS) data analysis. It is crucial to recognize the need for experience with Unix/Linux, either as a course pre-requisite or as a training need that can be addressed at the start of a course. Gathering such information from candidate participants in advance helps to identify this kind of training need [10].

#### Set learning objectives

Training needs should be perceived as such from both sides—by trainers as well as trainees. Therefore, explicitly mentioning the learning objectives (LO) of a course, or of a specific section of it, is strongly recommended. An LO is a clear statement of what the trainee(s) will be able to do as a result of the training, to what standards and under what conditions. LOs should be mentioned in the course description and designed in tune with participants’ backgrounds and capabilities. LOs should always be formulated in terms of competencies, using verbs like ‘reproduce’, ‘apply’, ‘predict’, ‘compare’, rather than ‘know’. This is because the former abilities can be translated directly into practical tasks and exercises, which represent essential tools to achieve LOs, whereas knowledge is related to principles, and it is usually acquired more indirectly through long-lasting experience or university courses.

### Matching training provided to audience

#### Selecting suitable trainees

Most training programmes and individual events are planned with the assumption of a particular training need in an, as yet, unknown audience. Prospective applicants will need to apply under one of a variety of possible mechanisms, from first-come-first-served to specific selection procedures. Matching the suitability of trainees to the training offered becomes a significant challenge in itself. For example, two potential trainees may need to know about NGS-data analysis: their end goals may be the same, but if one is a biochemistry researcher with an MSc in computational biology and the other is a clinical geneticist, they are likely to need to take different routes to achieving them. Therefore, whenever possible, it is recommended to define selection criteria that allow collation of applicant information, regarding: (i) relevance of the course topic to their scientific needs; (ii) their expectations about the course (e.g*.* are these realistic?); (iii) the suitability of the scope of the course to their career stage (e.g. are they well matched?); (iv) their fulfilment of course pre-requisites (e.g. can they program in Perl?). This information can be obtained by including a brief questionnaire in the course application form. When it is not possible to collect previous information about applicants, it may anyway be useful to do it at the start of the course, to have the possibility of adapting the teaching accordingly.

Of course, despite having followed these recommendations, it is still possible that a selected group of trainees may not fit a course perfectly, or may not be satisfied by it. This can happen for various reasons: not all trainees are capable of learning everything—some aspects of a course may simply be too difficult for them; some trainees might have been obliged to apply to a course to plug a perceived skills gap but find the course pitched at the wrong level; others may have been pressured to apply to fulfil the needs of their project, but find they have no genuine interest in many (or all) of the course topics. Situations like this rely on trainers’ sensitivity to detect these circumstances and to pay special attention to motivate such participants, e.g*.* by involving them in the solution of exercises before a class or giving them specific, tailored assignments, such as wrapping up at the end of the day or leading a brainstorming session.

#### Identifying appropriate trainers

Good trainers not only have appropriate subject knowledge but also good pedagogical and andragogical skills, are conscious of individual learning styles and paces and have the ability to ensure that participants interact and maintain their interest. Once the need for a specific training course has been identified, the organizer has to decide who will teach it. Unless the host organization has qualified trainers available, this is not an easy task. Indeed, there are no resources providing lists of recognized or accredited bioinformatics trainers, and most recruitment still occurs through personal knowledge of specific individuals, regardless of whether better trainers exist. A good candidate trainer is someone who is both expert in a topic and has experience of teaching it, whether in academia or in bespoke training courses. Generally, as the approach to short courses is fundamentally different from academic teaching, trainers with specific short-course experience may be more suitable than university professors. However, many good trainers have experience in both short training- and longer educational courses, and their teaching practice may be the richer for it.

Course organizers (individuals or institutions) represent a possible source of information when seeking appropriate trainers. Furthermore, for a course on a specific bioinformatics resource (database or tool), advice may be sought from the resource developers: often, they are able to provide specialized trainers or to organize courses themselves.

In an effort to make trainer selection easier, organizations like GOBLET (Global Organisation for Bioinformatics Learning, Education and Training) are working to collect and make available the names and competencies of experienced bioinformatics trainers without making value judgements. How to develop databases of, and effective rating systems for, trainers is currently a hot topic.

### Preparing the training

Bioinformatics training should be flexible to accommodate different types of content, course duration and trainee-learning speeds and skill levels. A common theme is the need to select a digestible amount of content and to prepare bite-sized chunks of training. Choosing appropriate teaching methods and preparing course materials are also part of the training groundwork.

#### Choosing the course format

Choosing the right format depends critically on striking the right balance between course duration, level and participant backgrounds. In deciding on a training format, it is worth considering: the trainer-to-trainee ratio, the number of participants, the time available, the facilities available and the experience and expectations of the trainees. Table 1 summarizes five formats commonly used by the authors and their pros and cons.

**Table 1:** Pros and cons of different training formats

Course format	Pros	Cons
Lecture + PC practicals	Easy to structure and prepare, even if done by separate persons. Lectures allow for easier face-to-face communication.	A strong topic connecting both parts is necessary, otherwise the lecture may be perceived too theoretical.
100% PC practicals	Best suited to self-learning groups with lots of material, and if the trainer takes the role of a coach rather than an instructor.	There is little room to cover extensive theoretical content. Direct communication may be limited because the PCs draw attention away from the other course participants.
Seminar with PCs ready	Groups of up to 10 people can switch between PCs and face-to-face teaching smoothly. Works best with a PC-free zone in the same room. Conference table with laptops also works.	Difficult with larger groups. The PCs pose a distraction to some extent.
Remote e-learning session	No travel costs; potential to train large numbers of people.	Requires highly motivated and independent trainees and well-prepared material. The plan is difficult to change on the fly.
Blended learning (combined teaching approach)	Potentially allows the disadvantages of all other approaches to be overcome.	Higher investment for course organizers, requiring more planning.

#### Diversity of training methods

In face-to-face training, a plethora of methods can be used to deliver a successful course. In our experience, three golden rules apply: (i) the trainer should present content in an engaging way; (ii) the trainees should be stimulated to think actively during exercises; and (iii) interaction and discussion should be encouraged.

How can these rules be translated into specific actions and choices? Trainers are constantly in search of effective training methods; most want to find an optimal balance among the many available options: showing slides, promoting discussions and interactivity, solving exercises together, stimulating individual work, asking trainees to present a topic (‘flip classes’), organizing games, telling engaging stories, working in groups and so forth. None of these activities alone is a guarantee of success, and various stakeholders have suggested that the most effective balance is achieved through multimodal learning [11]. The question is, are some approaches more effective than others?

Many bioinformatics trainers use slide-based lectures and live demonstrations for information transfer and practical exercises to reinforce learning. This format is not necessarily optimal for teaching a new competency, as opposed to simply transferring knowledge. Examples of teaching methods used successfully in bioinformatics training courses include: (i) use of case studies (reduces the complexity of the subject and tells a ‘research story’ to which trainees can relate); (ii) provision of teaching materials, such as manuals, glossaries, tasks and questions (help trainees to learn independently and lift some of the burden from trainers); (iii) explanation of algorithms using simplified models supported by board games, role-plays or pen-and-paper implementations [12, 13]; and (iv) discussions in groups, and with the entire class (brainstorming, gathering pros and cons, panel discussions and so forth). Incorporating a variety of such methods helps address differences in trainees’ preferred learning styles and learning paces and is more likely to be effective than traditional approaches. The balance between different modalities, however, may depend on a trainer’s attitude and capability (for example, an engaging speaker may be more successful spending time presenting content than in making trainees work in groups).

Overall, learning is a complex phenomenon [14]. Experience suggests that the most effective training approaches combine several styles, which may vary from one trainer to another and from one audience to another, and should be adapted to the training circumstances. Attention, motivation and basic skill levels of trainees also play fundamental roles [10, 11, 13].

#### Creating a training plan

A training plan is a scheme of the content, teaching method(s), goals and time allocated for each phase of a training session (Table 2). This makes it possible for both the trainer and trainees to monitor how the training is progressing, it helps maintain the pace, and it avoids getting stuck too long, say, on a specific problem raised by a single trainee. However, a training plan should be treated as a general guide, rather than a strict set of rules, and should not, therefore, be adhered to simply for the sake of it.

**Table 2:** Example training plan for a 90’ PyMOL tutorial

Phase	Content	Method	Time
Warm-up	Haemoglobin active site	Show sample image	2’
	What makes a good image?	Brainstorming	5’
Instruction	Basic recipe: seven steps to create molecular images	Single slide	5’
	Haemoglobin active site	Trainer explains task and time limit	5’
Practical	Create a picture of the haemoglobin active site	Trainees work in pairs, supported by learning cards	50’
Transfer	Gallery with resulting images	Q & A session	18’
Repeat	Seven questions on using PyMOL	Online multiple choice quiz	*

### Developing and maintaining training skills

Trainers have to maintain both their subject-matter expertise and their training skills. Subject-matter expertise can be supported by attending relevant seminars and conferences, reading articles and interacting with peers. Developing and maintaining training skills for trainers with no formal training qualifications (the vast majority) tends to rely on ‘learning by doing’ and can often benefit by shadowing experienced trainers and learning from them. For example, an effective strategy for improving their training skills is for trainers to sit in each others’ sessions, to observe their training practices and tricks, to consider what works and what does not and to incorporate successful modalities in their own sessions. Periodically participating in ‘train the trainer’ courses is another way to keep updated and to gain exposure to state-of-the-art training techniques. Other avenues are explored in Table 3.

**Table 3:** Some tips for developing and maintaining training skills

Skill	Where	Pros and cons	Website
Presentation skills	Public speaking blogs or clubs	They are helpful to improve presentation skills but are not specific for bioinformatics.	http://sixminutes.dlugan.com http://mannerofspeaking.org/ http://www.craigvalentine.com http://www. toastmasters.org
Andragogic knowledge	Educational literature and professional trainers	They are often expensive in time and/or money. Many professional courses focus on occupational learning and it can be hard to translate your learning to bioinformatics.	www.academis.eu/blog/tags/teaching/
Learning from peers	Communities of practice	Learning from your peers is inexpensive, effective and rewarding.	http://www.biotnet.org http://www. mygoblet.org/ http://www.iscb.org/iscb-education-committee

### Evaluating the training

Developing and running training events is a dynamic process. No training event will be perfect the first time and, with scientific technologies constantly in flux, courses need to adapt accordingly. To maintain and improve the quality of a course in the face of such fluctuations, it is important to assess whether the training offered was effective and to ascertain trainees’ perceptions. These two aspects need not necessarily coincide, as several other factors (some independent of training quality—the quality of the accommodation, the food provided and so forth) may influence satisfaction ratings.

It is important to review the course learning objectives and prerequisites, and participants’ prior- and post-event competencies. Collecting feedback is, therefore, essential. It is common practice to solicit feedback from trainees using an evaluation form. Paper-based forms have the advantage that they are quick to complete but the analysis takes longer. Online forms (e.g. using SurveyMonkey, http://www.surveymonkey.com) work well, provided sufficient time to complete them is scheduled towards the end of the course. For those who organize many courses, it is valuable to have a standardized form, allowing trainers and organizers to compare events. The option of completing the form anonymously may help to gather more honest opinions and ratings; regardless, participants should be encouraged to provide contact details—they may wish to be contacted to learn how their feedback has been taken into consideration.

The timing of feedback is also important, as there is a fine balance to be struck between asking for detailed comments at the end of a possibly lengthy and tiring course and allowing too much time to pass so that participants forget aspects that may be important. Short exit polls at the end of each day/session offer one way to gather key criticisms and positive features of individual sessions, with a more in-depth questionnaire provided in an allocated timeslot at the end of the full event or within a day or two of training completion. This contrasts with long-term feedback, which aims to identify the long-term benefits of a training course—this will be discussed later.

In many cases, a second source of reciprocal feedback is provided between the trainers and organizers. For the organizers, the aim is to gather suggestions on ways to improve the overall experience for trainers at future events (were there any problems with logistics, technical support, set-up of the room, equipment and so forth?) and to get an idea from the trainers about what they thought about the trainees (were they what was expected based on the pre-course data? Was the course properly targeted?). For the trainers, this is an opportunity to collect feedback on their training skills, and to get ideas and suggestions about content that could allow them to make improvements in future.

A third form of feedback is rarely collected: peer review. Here, trainers get direct feedback from another trainer or from someone able to assess the effectiveness of their training sessions. This is arguably the best way for trainers to receive the feedback they need to improve their training delivery, but it does require a relaxed and open/honest environment for it to succeed—trainers may be uncomfortable taking feedback from their peers, or may be uncomfortable providing feedback, no matter how constructive it may be. On the other hand, feedback from peer review may also be implicit rather than explicit. In other words, trainers who sit in each other’s sessions may assess for themselves the pros and cons of what they witness. This may lead to a kind of passive transfer of ‘good practice’, as they subsequently make conscious efforts to build into their training approaches new things that they perceived to work well, or to discard things that they perceived to be less successful. Such passive transfer can be more effective than direct peer assessment, which brings with it all the tensions outlined earlier in the text.

If peer review is not possible, or is difficult to provide, an effective way for trainers to identify improvements to their techniques is to video their own training sessions and review them after the event. Videoing a session is relatively low in cost (all that is required is a moderately efficient digital camera with sound) and allows an accurate analysis of the training sessions, but it may intrinsically affect the dynamics of the training and, to be effective, requires a great deal of self-motivation and an ability to analyse the recordings objectively.

#### Impact of feedback on future training

After collecting feedback from trainees, trainers and organizers, it is important to incorporate and respond to it to be able to improve subsequent similar events. The difficulty with such comparisons is that there are so many factors to consider that it is often difficult or impossible to pinpoint actual causal links to training improvements. Ultimately, though, the responsibility for maintaining and improving the quality of future training events lies with organizers, as they tend to have more holistic visions of their training programmes and tend to interact closely with multiple trainers across different topics.

Training is a skill that can be developed over time with suitable feedback and repetition. When facing points that need improvement, it is important to identify the real cause of the problem that is affecting training quality. This is much easier if trainers are open to applying new techniques and methods to their sessions and can be a fun process when trying out new ideas with an audience. One possible route to facilitate this process is simply for trainers to meet regularly to discuss problems and propose new solutions, or to prepare and deliver courses in partnership with other trainers. The use of standardized course-evaluation forms could also make it much easier to track trends and to measure the impact of feedback over time.

A common measure of long-term effectiveness is how many new collaborations course alumni were able to start as a direct result of training event. One possibility for long-term monitoring of bioinformatics training, with which some of the authors have now begun to experiment, is to use social media to keep in touch with course alumni. Another is asking participants to acknowledge training events in publications [15].

### Defining a sound organizational framework

The organizational aspects of a training event are of crucial importance for the outcome and, thus, for the satisfaction of trainees and trainers. It is, therefore, important to define the ideal conditions under which excellent training is possible. Crucial aspects to take into account are setting timelines, promotion, venue and IT infrastructure and support.

#### Setting timelines

Ideally, preparations for a new course targeting international trainees should start 12–18 months before the event. The venue, an outline of the course content and the trainers can be decided several months in advance. If participants are accepted on the basis of a selection process, it is worth closing the applications at least 2 months before the course, so that there is enough time to scrutinize all CVs and to inform accepted applicants in good time, so that they can arrange their travel. For local courses, with no restrictions on the number of participants (or with a pre-defined list of participants), a shorter deadline can be reasonable.

#### Promoting the training

There are no rigid rules regarding how much in advance a course should be announced and promoted. Three to 12 months beforehand is a reasonable and effective time frame if the course targets international trainees, otherwise a shorter period may suffice. It is advisable to use several different promotional channels and several time points. Moreover, it is important to specify a number of key event attributes or parameters: the course title, date, venue and organizers; the target audience, course prerequisites, learning objectives; a brief description of the content or a draft of the program; and the fees, if there are any (and what is covered by the fees). Although there is currently no accepted standard for what to include in course announcements, promising new initiatives, such as SASI (Scientific Announcement Standards Initiative for the life sciences), are beginning to emerge.

#### Choosing the venue

In choosing the venue, the highest priority is to verify the presence of a good IT infrastructure and relevant support. Internet access and any necessary software must be available and functional, and a person from the IT staff should be available during the course, if not constantly present in the room. At least a desktop computer or terminal for each two trainees should also be available. It is advisable to pre-configure computers with the same operating system, specific software and data sets required for the course. If participants are allowed to bring their own laptops, the venue must provide either Ethernet cables or wireless connection, sockets, adaptors and other equipment and space for the additional computers.

Furthermore, the venue should be large enough to accommodate all participants, trainers, computers, tables, projector and any other necessary materials; a whiteboard and/or flip board (with markers) may, in addition, be useful. It is also important to be able to secure the room during lunch and coffee breaks, and there should ideally be ready access to washrooms, catering and breakout spaces.

### A case study: training life scientists in programming and software development

Students choose to study life sciences for many reasons, including the difficulty of learning abstract and/or conceptually ‘hard’ disciplines, such as computer science. However, at some point in their careers, especially if they have to manage large data sets typical of high-throughput biology, they may ultimately need to manage their data with programming techniques, or to coordinate projects involving programmers. This may happen, for instance, with NGS data analysis, in projects requiring complex statistics or customized file parsing; or in life science projects that involve database creation, curation and server implementation.

Training life scientists in the rigours of statistical analysis, programming and database maintenance have increasingly become the responsibility of bioinformatics trainers. Experience shows that pure computational scientists tend to have less understanding of the particular needs of life scientists, of their backgrounds, of their ways of thinking and, crucially, in some cases, of their aversion towards programming. Indeed, the conceptual gulf between life scientists and computational scientists can be enormous, and bioinformatics trainers have become a key to bridge this gap.

One thing that trainers must keep in mind is that most life scientists wishing or having to embrace programming will not be interested in becoming developers. Thus, at least for beginners, it is advisable to teach the bare minimum of tasks needed to allow trainees to become independent in data analysis, such as file reading, parsing, manipulating and writing. More advanced language structures and tricks might be discouraging and even frightening. Collecting previous information about applicants becomes particularly important in this case. In fact, it makes it possible to adapt exercises to participants’ scientific backgrounds and needs: discovering that—with relatively few commands—they can solve complex problems, which they were unable to solve before, can be extremely motivating.

It is also good practice to allow trainees to work in pairs on focused tasks. This not only tends to speed problem solution but also encourages discussions and shows that different programmers can have diverse, equally effective, styles. Another important point is the value in having trainers explaining how to translate biological questions into programming tasks. Programming is just a different way to ask questions and to solve problems, something scientists do all the time. Becoming aware of this can help bring down the barriers to or resistance towards code writing that is so often experienced by life scientists.

As already mentioned, virtually all life scientists who decide to learn how to program will not become software developers. Nevertheless, it is advisable to introduce them, from the outset, to best practices in software development, such as the Agile and the Software Carpentry philosophies [16–19], designing projects upfront, version control systems [20, 21], debugging techniques [22] and so on. In this regard, trainers have massive responsibilities: although such practices are natural to computer scientists, they remain almost unknown to bioinformaticians originating from the life sciences, despite being the only guarantees of efficient programming and of trust and reproducibility of results. These practices are also crucial if code writers are to collaborate effectively with one another. Many legacy problems in the field of bioinformatics result directly from the failure of previous generations to adhere to these practices; the imperative for bioinformatics trainers today to encourage and promote adherence to good programming practices during their courses is consequently ever more urgent.

## CONCLUDING REMARKS

Demand for bioinformatics training is increasing as more and more life scientists, working in diverse research areas, are generating and using data produced by high-throughput methodologies. To date, bioinformatics training has developed rather organically. In Europe, a handful of organizations (some publicly funded, some companies) that offer user-training programmes exist, but for the most part, training is organized in an *ad hoc* way in response to local demand, often using local trainers who are bioinformatics experts but have little or no training experience. By sharing experiences of what works and what does not, it is possible to develop best working practice and improve the overall quality of bioinformatics training.

This article summarizes the key discussion points from a more extensive best-practice document on bioinformatics training for life scientists. This is much a living document that will continue to evolve as training methods evolve, and as experiences from a broader community of professionals share interests, ideas and experiences. To help crystallize and further galvanize this community of trainers, several organizations, networks, societies and individuals around the world have become members of the new Global Organisation for Bioinformatics Learning, Education & Training (GOBLET, www.mygoblet.org), to facilitate this on-going dialogue and to share training materials, experiences and best practice worldwide.

**Key points**

- Demand for bioinformatics training is increasing tremendously, largely owing to high-throughput data generation and the need for robust data analysis.
- In this context, achieving excellence in training is a considerable challenge.
- Here, we discuss training excellence and how it might be achieved.
- We suggest working practices to identify training needs, to articulate learning objectives and to ensure delivery of suitable training for given audiences, a quality-assurance process and a sound organizational framework.
